# A protocol for co-creating research project lay summaries with stakeholders: guideline development for Canada’s AGE-WELL network

**DOI:** 10.1186/s40900-020-00197-3

**Published:** 2020-05-08

**Authors:** Mineko Wada, Judith Sixsmith, Gail Harwood, Theodore D. Cosco, Mei Lan Fang, Andrew Sixsmith

**Affiliations:** 1 Science and Technology for Aging Research (STAR) Institute, Simon Fraser University, #2800 – 515 West Hastings St., Vancouver, BC V6B 5K3 Canada; 2 School of Nursing and Health Sciences, University of Dundee, 11 Airlie Place, Dundee, Scotland DD1 4HJ UK; 3 411 Seniors Centre Society, #7th Floor – 333 Terminal Avenue, Vancouver, BC V6A 4C1 Canada; 4 Department of Gerontology, Simon Fraser University, #2800 – 515 West Hastings St., Vancouver, BC V6B 5K3 Canada; 5 Oxford Institute of Population Ageing, University of Oxford, 66 Banbury Road, Oxford, OX2 6PR UK

**Keywords:** Guidelines, Lay summary, Co-creation, Collaborative team research, Stakeholder involvement

## Abstract

**Background:**

Funding bodies increasingly require researchers to write lay summaries to communicate projects’ real-world relevance to the public in an accessible way. However, research proposals and findings are generally not easily readable or understandable by non-specialist readers. Many researchers find writing lay summaries difficult because they typically write for fellow subject specialists or academics rather than the general public or a non-specialist audience. The primary objective of our project is to develop guidelines for researchers in Canada’s AGE-WELL Network of Centres of Excellence, and ultimately various other disciplines, sectors, and institutions, to co-create lay summaries of research projects with stakeholders. To begin, we produced a protocol for co-creating a lay summary based on workshops we organized and facilitated for an AGE-WELL researcher. This paper presents the lay summary co-creation protocol that AGE-WELL researchers will be invited to use.

**Methods:**

Eligible participants in this project will be 24 AgeTech project researchers who are funded by the AGE-WELL network in its Core Research Program 2020. If they agree to participate in this project, we will invite them to use our protocol to co-produce a lay summary of their respective projects with stakeholders. The protocol comprises six steps: Investigate principles of writing a good lay summary, identify the target readership, identify stakeholders to collaborate with, recruit the identified stakeholders to work on a lay summary, prepare for workshop sessions, and execute the sessions. To help participants through the process, we will provide them with a guide to developing an accessible, readable research lay summary, help them make decisions, and host, and facilitate if needed, their lay summary co-creation workshops.

**Discussion:**

Public-facing research outputs, including lay summaries, are increasingly important knowledge translation strategies to promote the impact of research on real-world issues. To produce lay summaries that include information that will interest a non-specialist readership and that are written in accessible language, stakeholder engagement is key. Furthermore, both researchers and stakeholders benefit by participating in the co-creation process. We hope the protocol helps researchers collaborate with stakeholders effectively to co-produce lay summaries that meet the needs of both the public and project funders.

## Plain English summary

Funding bodies often require researchers to write lay summaries (summaries in non-scientific language) to share their research with the public and explain its importance. However, researchers typically find lay summaries difficult to write and the public finds them difficult to read. If stakeholders outside the academic sector are involved in writing lay summaries, the summaries are more likely to be understood by the public. Our project aims to develop guidelines for researchers in Canada’s AGE-WELL Network of Centres of Excellence, and eventually in various other disciplines, sectors, and institutions, to help them work with stakeholders to co-create lay summaries. We have created a lay-summary co-creation protocol based on workshops conducted with an AGE-WELL researcher. This paper presents the protocol, which researchers funded by the AGE-WELL Core Research Program 2020 will be invited to use to work with a range of stakeholders to co-produce a lay summary of their projects. The lay-summary co-creation protocol has six steps: Learn the basic steps for writing a good lay summary, identify the target readership, identify stakeholders to work with, recruit stakeholders to work on a lay summary, prepare for workshop sessions, and run the sessions. To help researchers get the most from their experience, we plan to give them a guide to writing a *good* research lay summary, help them make decisions, and host, and facilitate if necessary, their lay summary co-creation workshops. This protocol would help researchers write effective lay summaries to share their research with a wide group of readers.

## Background

A lay summary is a brief synopsis of a research project that explains in plain language its essential components—what, who, where, when, why, and how—to the general public or a target, non-specialist audience [1, 2]. It is imperative to develop a lay summary and make it available to the general public for several reasons. First, a lay summary provides a way to communicate to project funders what issue or problem a project aims to solve, why it is important to address the issue or problem, how the researchers aim to solve it, and how the funding will be used in the project. The lay summary also enables members of the public on a funding application review committee to be fully included in the decision-making process and allows the researchers to demonstrate their accountability to the funding bodies [3–5]. Second, rather than being limited to a niche, perhaps academic or specialist, group, a lay summary increases the visibility of a project because it can be made more universally accessible, thereby creating a broader readership and increased awareness of and understanding about the issue that the project aims to address [3, 4, 6, 7]. However, it should be noted that “accessible” is a relative term and so it must be defined and applied in the specific context of a project and in terms of the aim of the text and its target readership [8]. Third, a lay summary enables researchers to communicate the real-world relevance of their projects’ implications to the public, and as such, funding bodies are increasingly urging researchers to produce lay summaries that will help improve public awareness and understanding of projects and their overall importance and impact on everyday life [3, 9, 10]. Fourth, in the case of projects that require recruitment of participants, a lay summary can help potential participants understand the study and its goals and decide whether or not to participate [3, 4]. Finally, a lay summary that stems from a health or medical study or intervention can inform patients’ decision-making on medical and pharmaceutical interventions, which will help facilitate the adoption of project outputs that can solve the target issue, such as the development or use of a technology product or services [3, 11, 12].

Many researchers are facing increasingly frequent requirements from funders to develop lay summaries, but even those who recognize the benefits of doing so often find the process challenging and cumbersome primarily because they are immersed in an academic scholarship environment. They are thus conditioned to use traditional academic communication channels (via research proposals, peer-reviewed articles, and conference papers and posters) and specialized academic language [6, 7]. Academic writing frequently draws heavily on jargon and other subject-specific terminology, which can come across as difficult or opaque to lay readers. Using academic writing practices to disseminate newfound knowledge is therefore fundamentally exclusionary and runs counter to the current movement towards meaningful stakeholder participation, inclusive research, and co-creation methods. If academic/research institutions, researchers, and funders are claiming to strive for equity, diversity, and inclusion and want those claims to be taken seriously, they must communicate information in an open and accessible way to enhance inclusivity. This requires a significantly different approach to traditional methods of communicating research, not only in terms of how the research is presented (language level, amount of detail, medium of communication, for example) but also in terms of who is involved in the process of creating (writing) the information (e.g., stakeholders with limited knowledge of current developments in science and state of the art research). However, there appears to be a certain reticence among researchers to undertake a more collaborative model of research, possibly because of a reluctance to deviate from prevailing traditional academic expectations and cultures [13]. Until researchers both understand the benefits of producing and using a lay summary and master the relevant processes and techniques involved, they may perceive this requirement as an unnecessary burden [3] or as another barrier to publishing peer-reviewed papers [7] or gaining research funding.

### Collaborative team approach: stakeholder involvement in the research process

Patient and public participation is an increasingly popular approach to research [14], and a collaborative team approach is proving to be essential in various fields of research and practice [13, 15–17]. The collaborative research approach involves academics/scientists collaborating with stakeholders from different disciplinary and sectoral backgrounds (e.g., older adults, caregivers, community organizations, industries, policymakers) to foster knowledge exchange and integration across disciplines and sectors [18]. This process of cross-disciplinary and cross-sectoral knowledge exchange and integration promotes a co-production of knowledge that transcends disciplinary and sectoral boundaries [15, 18]. Such an approach is essential for understanding and addressing complex, real-world problems because they are context-specific, needs-driven, and multifaceted, all of which renders the implementation of a traditional, uni-disciplinary approach inadequate [15, 18]. The collaborative team approach requires researchers to work with stakeholders as research partners from the outset to identify a real-world problem, understand the multilayered issues that surround it, co-develop project objectives, and co-design and implement the project [18].

The process of creating a lay summary is an inherently collaborative one: ensuring that researchers work on an equal footing with key stakeholders at an early project proposal stage is critical to their fully understanding each stakeholder’s language ability and literacy level, as well as their interest in, experience of, and knowledge level of an issue that a project aims to address [11, 19]. In particular, incorporating lay perspectives into research is perceived as beneficial for facilitating understandings of problem areas and increasing a research team’s capacity to generate more effective solutions [13, 20, 21]. The integration of lay perspectives is also politically mandatory, as the general public technically own publicly funded research and are entitled to have their voices heard and to legitimize decisions [22]. Lay people who are involved in research as research partners can also benefit directly from their involvement, as demonstrated by Duke [14] and Wada and colleagues [13]. Both studies identified empowerment, social engagement and connectedness, inclusivity, and skills and knowledge development as potential benefits that lay people can gain through involvement in research projects.

While general guidelines for writing a lay summary tend to provide tips on content and word choice [23], few of them contain sufficient detail to expand researchers’ understanding of how to create a lay summary that communicates scientific knowledge effectively to the general public or a non-specialist target readership [9, 24]. In addition, while collaborating with key stakeholders is often critical for developing a lay summary that is written in accessible language and includes information that is relevant to the target readership, to date there have been few guidelines that can help researchers navigate the process of co-creating a lay summary with stakeholders [12].

### Project context

This project is part of an ongoing priority in AGE-WELL NCE (Aging Gracefully across Environments using Technology to Support Wellness, Engagement and Long Life), a Pan-Canadian Network of Centres of Excellence (NCE) focusing on aging and technology. In 2019–2020, AGE-WELL funded 24 projects focused on developing technology-based solutions to address issues experienced by older adults and caregivers. As AGE-WELL explicitly advocates a focus on real-world impacts and the implementation of collaborative team research within the network, there is a growing need for guidelines to help researchers co-develop key outputs of their projects with stakeholders, such as lay summaries of their projects. Accordingly, the primary objective of our project is to develop lay summary co-creation guidelines for AGE-WELL researchers that will ultimately be made widely available to researchers across a variety of disciplines, sectors, and institutions. We began this project by producing a protocol for co-creating a lay summary that consists of a series of steps based on feedback and reflections from an AGE-WELL researcher and older adults who attended co-creation workshops we organized and facilitated for the researcher. The protocol was therefore a collaborative effort. This paper aims to present the lay summary co-creation protocol that 24 AGE-WELL–funded researchers will be invited to use.

## Methods

### Participants

Eligible participants will be researchers who have funding for AGE-WELL’s Core Research Program 2020 (CRP).

### Recruitment

We will recruit participants by sending an email to the 24 CRP researchers. It will describe the aims of the project, include a guide to creating a good lay summary of research projects, and provide contact information for the principal investigator of the project.

### Co-creating lay summaries of research project with stakeholders: protocol

**Fig. 1:**
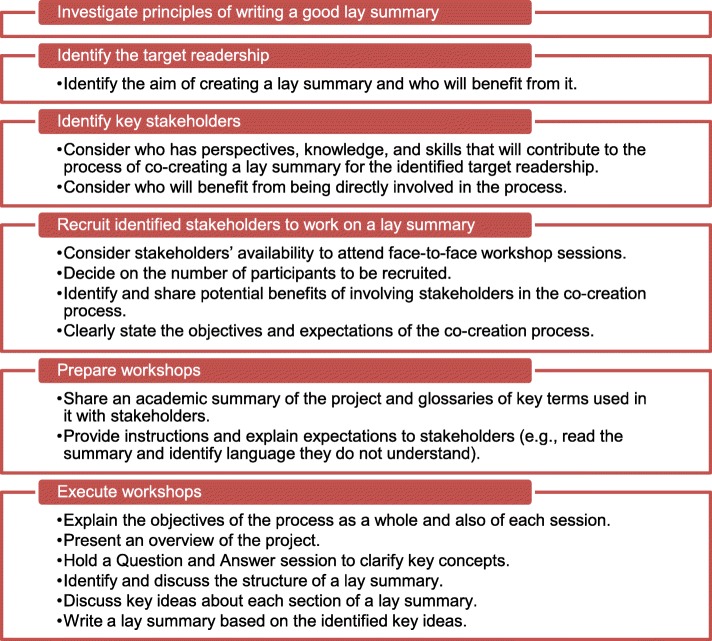
Process of co-creating project lay summaries with stakeholders

Figure 1 illustrates the protocol for co-creating project lay summaries with stakeholders: investigate principles of writing a good lay summary, identify the target readership, identify key stakeholders, recruit them, prepare for co-creating lay summary workshops, and execute the workshops. We will guide participants in navigating the lay-summary co-creating process via one-on-one consultations. In particular, once participants have identified the target readership for their respective lay summaries, we will host, and facilitate if needed, workshops with them and their identified stakeholders to enable the co-production of lay summaries of their CRP project.

#### Investigate principles of writing a good lay summary

**Fig. 2:**
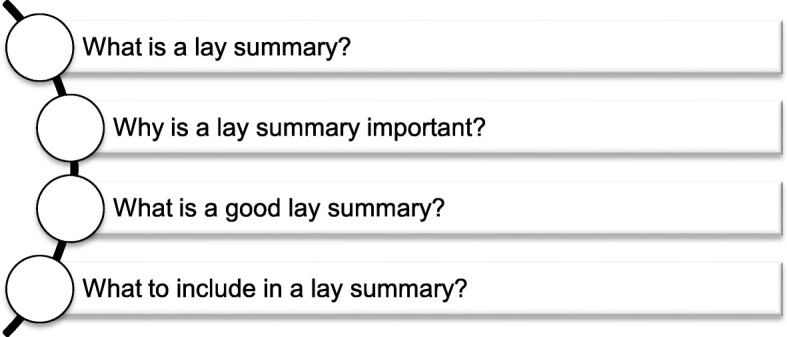
Four components of the guide to developing a good lay summary of research

We will provide a guide to developing a good lay summary of research (Additional file 1) to 24 participants once they have agreed to be involved in this project. The guide comprises four sections (Fig. 2). Drawing from the literature on and available guidelines for developing a lay summary, we include a definition of a lay summary and explain why it is important to create one. We also emphasize that identifying the target readership is a critical step as it informs participants about what information needs to be included in a lay summary and what language, or literacy, level the lay summary needs to be written in. Additionally, the guide explains the basic principles of writing a good lay summary, with a particular focus on precision and succinctness and the use of plain language. The guide also contains five questions that should be answered in a lay summary—1) What problem needs to be addressed? 2) What are the aims of the project? 3) How will the project be carried out? 4) Why is the project important? and 5) What are the expected outcomes or impacts of the project?—and includes examples of the responses to these questions.

#### Identify the target readership

Participants will identify the target readership for their lay summaries. While a lay summary is generally targeted at the general public, it is also broad in scope [6]. Defining the readership at the outset is critical to the development of an accessible and compelling lay summary because it determines the level of interest in an issue that a project seeks to address, the knowledge level about the issue, the language, or literacy, levels of the target readership, and the ultimate format of the lay summary [3, 11, 25]. For example, guidelines for target reading levels in lay summaries vary from Grade 6 to Grade 10 reading levels [4, 11, 26]. However, while a Grade 8 reading level is standard for newspapers and is thus often seen as offering a general assessment of reading levels among the general public [11], Nunn and Pinfield [5] note that reading levels vary among readerships and that readers with a high literacy level tend to be the group that benefits most from a lay summary. This means that a large segment of the general public will potentially not be in a position to benefit from a lay summary. To minimize this risk, it is therefore important to think carefully about who the target readership is, whose perspectives need to be integrated into a lay summary (e.g., older adults, caregivers, funders, researchers), and why the lay summary is being created (e.g., to recruit participants in a project, to increase awareness of an issue to be resolved in a group of people who experience the issue).

#### Identify stakeholders to collaborate with

Keeping in mind the target readership identified, participants will then decide which stakeholders to collaborate with to create their project lay summaries. Essentially, some stakeholders involved in the co-creation process should represent the target readership of the lay summary, because they are the most likely group to provide pertinent insight and perspectives. It is therefore important to have a clear understanding of what type of perspectives and expertise will be of most value to a lay summary in terms of its meeting the needs of the identified target readership. Furthermore, taking into account who will benefit from being directly involved in the process (e.g., empowerment, awareness of the project and its aims), and how they will benefit (e.g., increased knowledge about potential solutions), might help participants identify which stakeholders to work with. Ideally, participants and key stakeholders should collaborate at every stage of developing the lay summary to optimize participants’ opportunities to spontaneously learn, discuss, negotiate, and integrate different perspectives. This level of collaboration facilitates an iterative and more organic co-creation process, and thus the resulting lay summary is more likely to be fit for purpose and mutually agreeable.

#### Recruit identified stakeholders to work on a lay summary

Once participants have identified who to collaborate with in co-creating a lay summary, they start the recruitment process. This step can be a challenge, as lay-summary co-creation requires particular time commitment from stakeholders. Participants must ensure the identified stakeholders will be available to attend any face-to-face co-creation sessions, but family caregivers, for example, may have difficulty attending sessions during the day unless they can find someone to replace them at home. Participants must therefore be prepared to be flexible in terms of the formats and timings for meetings for the co-creation process.

Participants must give careful consideration to the optimum number of stakeholders to recruit for the co-creation process. A group of 6–10 with relatively diverse backgrounds may introduce different ideas and perspectives but reaching a consensus could prove challenging. In contrast, a group of 2–4 may result in more focused discussion and streamline the co-writing, co-revising, and co-editing process, but the scope of diversity is necessarily more restricted.

Recruitment methods will vary. A multi-methods approach can be effective as it addresses people’s different preferences for and accessibility to various communication methods (e.g., flyers, emails, social media platforms). Approaching community organizations and groups to which target stakeholders are likely to belong and asking them to circulate recruitment notices to their members (e.g., seniors’ centre, patient groups, family caregiver associations) would increase the number of target stakeholders that participants can reach out to. Regardless of which medium is used, all the objectives and expectations of the project need to be clearly and concisely expressed. For instance, participants may state the overall objective of the co-creation process: “To write, in simple language, a summary of a project that investigates how family caregivers manage giving medication to older adults living with dementia.” If the process extends over multiple sessions, the objective of each session may be described: “The first session focuses on developing a shared understanding of the project” and “the second session focuses on discussing a drafted lay summary and finalizing it.”

#### Prepare workshop sessions

Participants will prepare for their lay-summary co-creation workshops by making decisions about the number of co-creation workshop sessions they plan to have, and the objectives of each session. The number of sessions that will be needed to complete a lay summary should be calculated based on the availability, skills, and experiences of the participating stakeholders. For example, if a wide range of stakeholders are invited, and includes stakeholders who do not consider themselves to be writers in any way, the writing process may take longer.

Participants will identify and explain the objectives of each session to stakeholders prior to the workshop. For example: “The first session aims to develop a shared understanding of a project, and the second session will focus on writing, revising, and/or editing a lay summary.” For each session, participants will plan what instructions and guidance they will give to stakeholders before they come to sessions so that both groups can make best use of their time. For example, participants will consider 1) sharing an academic research proposal or the original summary prior to the first session with the stakeholders, and 2) developing and sharing glossaries of key terms used in the original research proposal and summary. It is also important to inform stakeholders what they are expected to do prior to the sessions (e.g., read the original summary, identify language they do not understand, and be ready to discuss the summary in a session) as well as during the sessions.

#### Execute workshop sessions

**Fig. 3:**
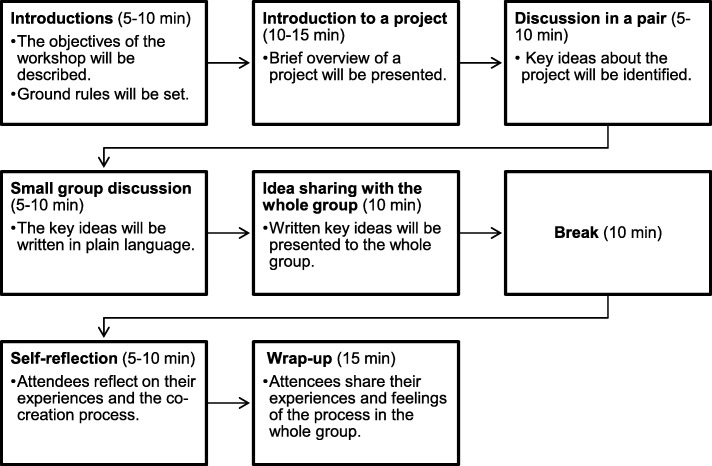
An example of a first workshop session

A participant may start a session by welcoming stakeholders, setting ground rules and expectations for the session (e.g., respect for different perspectives and ideas), and briefly explaining the objectives of the co-creation process as well as of each session (if there will be more than one session). The participant may then present an overview of their project to the stakeholders, followed by a Q & A about it. During the Q & A phase, stakeholders may request further clarification of key concepts of a project, which may lead to discussion about potential simple terms to describe them. After the Q & A, a participant may introduce and discuss the structure of a lay summary (e.g., problems/challenges, objectives, methods, and impacts). Key ideas about each section of a lay summary will be discussed. Small group activities may be a more effective approach for identifying key ideas to be included in a lay summary if stakeholders are introverted or otherwise hesitant to offer an opinion. A participant may end the session by sharing experiences of the co-creation process among the stakeholders. Throughout the session, a participant may have a facilitator present to help not only with time management but also with discussing and developing ideas put forward by stakeholders and a participant. Figure 3 presents an example of a first session.

Writing a lay summary based on the identified key ideas may be done in a subsequent session. Alternatively, at the end of the first session, the participant might invite stakeholders to draft a lay summary outside the scheduled sessions, although this is dependent on stakeholders’ motivation, availability, and skills.

### Ethical procedures

Ethical approval is not required for this project because its objective is to improve the quality of services and resources for promoting and supporting collaborative team research for researchers within the AGE-WELL network. According to Ethical Conduct for Research Involving Humans, quality assurance and quality improvement studies do not constitute research and thus do not require a Research Ethics Board review [27].

## Discussion

A lay summary is one example of the types of public-facing research outputs that are becoming increasingly important to project funders and the general public. This paper has presented a protocol for co-creating lay summaries of research projects with stakeholders based on lay-summary co-creation workshops we conducted with one AGE-WELL researcher. Reflections and feedback from the researcher and stakeholders who participated in the workshops on the co-creation process have been incorporated into the protocol. A catalyst for the development of the co-creation protocol was the recognition that many lay summaries published to date are not fit for purpose. It is a challenge to produce lay summaries that include information that will interest a target readership and are written in accessible language [3].

Initial feedback on the lay summary protocol suggested that researchers—and by association, projects—might not have the capacity to engage in the co-creation of lay summaries. However, this argument is becoming less relevant as collaborative team approaches and meaningful involvement of stakeholders increasingly become standard practice*.* The creation of lay summaries should not be treated as a necessary evil that gets tacked onto the real part of the research; it is an essential knowledge translation activity. Stakeholder engagement is critical for project planning and so should be adequately resourced in the same way that the “core” aspects of a project are planned and resourced. This may also include compensation and reimbursement for lay people who are involved in the co-creation process.

In terms of resources, funders and researchers need to consider the benefits—or added value—of co-creating lay summaries. A key aim of the development of our protocol is to position the production of lay summaries as part of a co-creation approach to research, particularly in the early stages of a project when the members of a project group are developing a shared understanding of its aims, approaches, and methods. This forces the researchers to think in terms of the target group whose issues they aim to address and engages the whole team (researchers and stakeholders) in reflecting critically on the ideas, objectives, and methods of the project. The production of lay summaries could be seen as a milestone and deliverable from the first stage of the co-creation process and as a way of building relationships and mutual trust within a team. It is envisaged that the project group will continuously update the lay summary, as well as co-create other public-facing outputs. Co-creation benefits researchers and funders as it fosters the production of more effective solutions to real-world problems through cross-disciplinary and cross-sectoral knowledge integration [13, 15, 18, 20, 21, 28].

The benefits of participating in the process of co-creating lay summaries may be as important as those offered by the final lay summary. Our experience in developing the protocol led us to identify several potential benefits of participating in the process:

- It can foster an effective working relationship within the project team (researchers and stakeholders).
- It can help both researchers and stakeholders better understand the co-creation process because the lay summary co-creation is an early stage of the overall co-creation process that will be applied throughout the lifetime of a project.
- It can validate the roles and contributions of stakeholders in a project, thus awarding them a greater sense of accomplishment.
- It can help researchers critically evaluate their ideas and proposal, prior to the main research phase.

### Strengths and limitations

Last, it should be noted that the protocol we developed is not without limitations. First, it focuses on supporting researchers as they navigate their way through co-producing lay summaries with stakeholders, and thus may not be useful to other groups of people who intend to develop lay summaries with stakeholders (e.g., funders, community organizations). Second, the protocol is a work in progress and its effectiveness needs to be evaluated. Despite some of the gaps, a key strength is that it offers one of the first step-by-step guides for researchers to co-produce project lay summaries with stakeholders. We hope that it will also serve as a tool that helps researchers not only to recognize the multiple values of collaborating with stakeholders but also to produce lay summaries that benefit both the public and project funders.

## Supplementary information

**Additional file 1.**

## Data Availability

Not applicable.
