# Supplementary material for: A protocol for co-creating research project lay summaries with stakeholders: guideline development for Canada’s AGE-WELL network
Source: Res Involv Engagem. 2020 May 8;6:22. doi: 10.1186/s40900-020-00197-3 (PMC7210667; doi:10.1186/s40900-020-00197-3)
Supplement: Supplementary file 1 — Additional file 1. [file 40900_2020_197_MOESM1_ESM.docx]

**Guide to Creating A Good Research Lay Summary**

While funding bodies are increasingly requesting lay summaries for research proposals to communicate projects’ real-world relevance to the public and support general public engagement in research [1], many researchers find the process difficult because they generally write for fellow subject specialists or academics [2,3].

While general guidelines for writing a lay summary are available [4], they are often inadequate to expand researchers’ understanding of how to create a lay summary that communicates scientific knowledge effectively to the general public or a non-specialist target readership [5]. The AGE-WELL Transdisciplinary Working (CC3) team therefore organized two lay-summary co-creation workshops for the AGE-WELL network and developed a guide to creating a good research lay-summary.

# What is a lay summary?

A lay summary refers to a brief synopsis of a research project that explains in simple terms its essential components—what, who, where, when, why, and how—to the general public or a target, non-specialist audience [6].

# Why is a lay summary important?

It is important for researchers to develop a lay summary of their project for the following reasons:

- Communicating to funders of a project what issue a project aims to solve, why it is important to address the issue, how it is to be solved, and how the funding allows the researchers to demonstrate their accountability to the funding bodies [1].
- A lay summary increases the visibility of a project, which in turn draws the attention of the general public or a target population to it and raises their awareness of and understanding about the issue that the project aims to resolve [1–3].
- In the case of projects that need participants, a lay summary can help potential participants understand the study and its goals and decide whether or not to participate [1].
- A lay summary facilitates the adoption of outputs that can solve the target issue, such as a technology product and services [1,7].

# What is a good lay summary?

## Be precise and succinct

The current general guidelines on writing lay summaries note that lay summaries should be written succinctly and clearly as possible [4]. While the length of a lay summary vary depending on the purposes and organizations that require it [3,7], it is typically 100-250 words for scientific journals [8–10].

- Ideas should be expressed in the active voice and phrased positively (avoid the use of not where possible) [1,11].


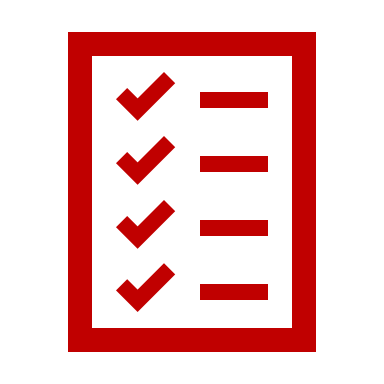


- Place a subject and verb close to each other at the front of a sentence [10].
- Use metaphors, images, comparisons and analogies to help visualize complex ideas [10].
- Using graphics and tables can be effective to present content [12]. Bulleted lists can be helpful [12].
- Simplify the sentence structure as much as possible [1,7]. Avoid long sentences and paragraphs by removing extra words wherever possible, but sometimes plain language will require more, rather than fewer, words to aid comprehension.
- Avoid too many parentheses, commas, and semicolons [13].
- Text should be organized logically to flow naturally [1].
- Using “respectively,” “former,” and “latter” can confuse some readers, and inconsistent use of terminology can frustrate readers [13].
- The word “may” can be ambiguous; use “can” or “might” instead.

## Use plain language

Researcher should use plain language (sometimes referred to as clear language) to make a lay summary accessible to a non-specialist readership [3,6,9,13]. Plain language is defined as writing that a target population can understand when they hear or read it for the first time [13].

- The use of scientific jargon, technical terms, and abbreviations should be avoided if possible, and at the very least minimized.


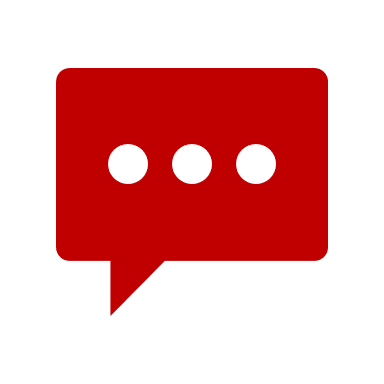


- However, it is important to recognize that common expressions can have specific meanings in certain contexts and are therefore at risk of being misinterpreted if they are not explained [7]. Thus, it is helpful to use and explain potentially ambiguous words or expressions in contexts where the readership is likely to encounter them (e.g., medication labels) [6].
- It is important to remember that oversimplifying words and phrases can increase the risk of lay readers feeling patronized, so it is important to aim for a balance between explaining and being respectful. Oversimplifying a lay summary can also take an opportunity to learn about problems of interest and solutions from target readership [12].

Furthermore, the design of the final document is important. A serif typeface, at least 10 points in size, helps readers move through a text. The page should have plenty of white space so that it is not overcrowded. Finally, the title should be short and to the point, with no abbreviations or technical terms [1,5,10].

# What to include in a lay summary?

While the general guidelines suggest that a good lay summary address the what, who, when, where, why, and how of a project [1,3], what to include in a lay summary depends on the needs and interests of the target readership [9,12]. We have developed a template that suggests including five components in a defined order in a lay summary (see Figure 1).

Figure 1. Outline of a lay summary of a research proposal

## What is a problem that needs to be addressed?

Introducing a problem that a project aims to address right at the beginning of a lay summary is an effective means of drawing the attention of the target readership. A good lay summary should therefore open with basic information—for example, what dementia is and how it affects people’s daily lives [4].


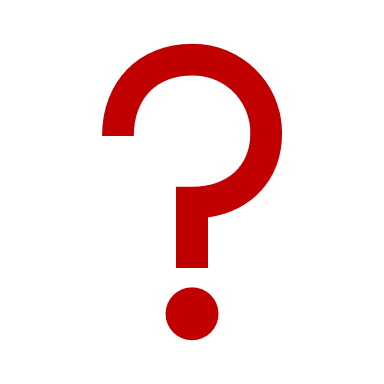


In the opening sentences, it is critical to introduce and describe in simple language key concepts of a project, such as dementia, social isolation, and cognitive decline. A problem that a project aims to address needs to be presented in the context of daily life. Instead of referring in general to memory loss, for instance, citing difficulty managing medication, missing doctor’s appointments, or forgetting to turn off the oven helps readers imagine and more easily relate to the problem of memory loss.

## What are the aims of a project?

After the specific problem has been described, the goals of the project need to be stated. The goals need to address the problem in a relatable way. For example, if a project focuses on medication management for community-dwelling older adults living with dementia, one goal may be to explore what tools and equipment the older adults have used and the advantages and challenges of using them.


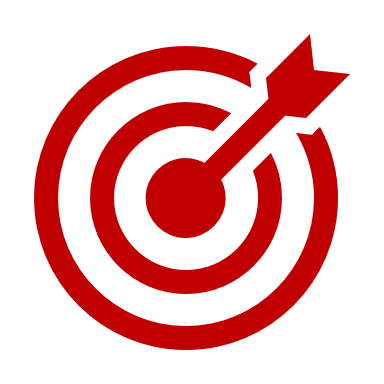


## How will the project be carried out?

The lay summary should describe clearly and briefly how a project will be carried out, including:


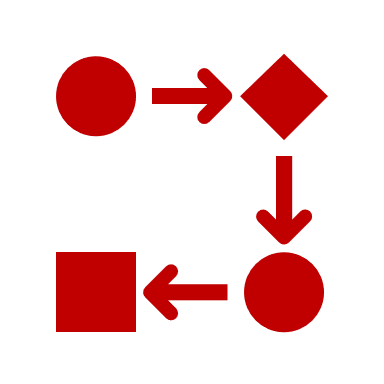


- What the process will be;
- Who will be involved;
- What information will be collected;
- Where the project will be conducted; and
- How the collected information will be used.

It is important to avoid using technical terms to describe the protocol of the project data collection and analyses. Lay readers may also interpret strategies and inclusion and exclusion criteria for participating in a project as unnecessarily selective. Thus, researchers should carefully select and list the criteria that are observable to the target readership and essential for the project—such as sex, age, physiological symptoms, or physical functioning [5,14].

## Why is the project important?

Explain why the project is important. Explain why achieving the stated objectives of the project is important. Link the rationale of the project to the problem described at the beginning of the lay summary. For instance, explain why it is important to investigate the tools and equipment that people living with dementia have used to cope with the problem of medication management and what advantages and disadvantages they experience in using them. Think about how the project can contribute to addressing the problem. Make the rationale relevant to the readership—for example, present it in terms of preventing further health issues, facilitating early detection of medical conditions, improving quality of life, decreasing caregiver burden, or developing new interventions or programs [4].


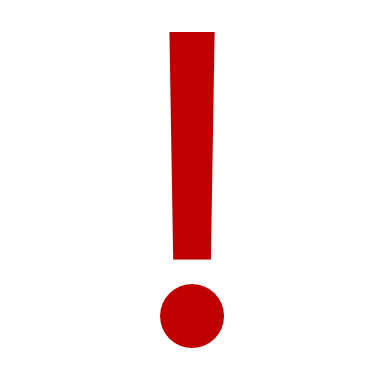


## What are the expected outcomes or impacts of the project?

Explain the expected impacts of the project in a broader context. How can it contribute to the economy, society, and science?


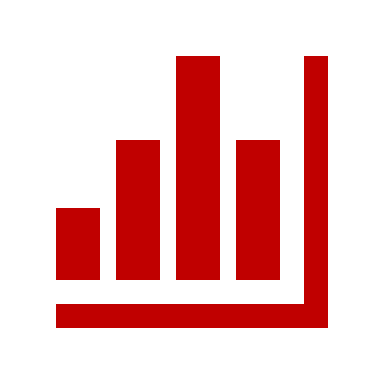


- Economic impacts include, for example, a reduction in care costs and increase in demands for technological services and resources to help people with dementia manage their medication.
- Social impacts may be a reduction in the demands on family caregivers and an increase in their quality of life.
- Science impacts can include creating opportunities for the development of technologies that support medication management for people with dementia.

Box 1 shows the original academic summary and a lay summary co-developed in the workshop.

Box 1: Example of how the original academic abstract (by Theodore Cosco) was transformed into a lay summary through the co-creation process

**Original abstract**

**Title:** **Precision mental health: A stakeholder-informed, Big Data approach to psychological wellbeing**

Mental illness is common and destructive. Worldwide, poor mental health directly affects one in four people, with many more experiencing negative physical health outcomes due to the impact of mental distress on non-psychological ailments. In order to address these challenge, it is imperative that older adults’ mental health perspectives and priorities are incorporated into research aims and study designs. We aim to disrupt traditional psychiatric approaches to mental health, by using stakeholder priorities as research outcomes rather than strictly using biomedical diagnostic criteria for mental disorders. Computational modelling of these priorities will inform a risk assessment platform that identifies individuals at risk for poor mental health. Precision medicine approaches take into account differences in individuals' unique characteristics (beyond the scope of traditional medicine) to treat and prevent illness. Many precision medicine disciplines have been highly successful, for example, precision oncology; however, precision mental health is under-studied and under-utilized. Using Big Data resources focusing on older Canadians, we will train a precision mental health model to predict the degree to which individuals are vulnerable to poor mental health. Application of these models in community partners’ real-world databases will enable the early identification of at-risk clients/residents/patients. In addition to risk profiling, we will also use quasi-experimental computational modelling techniques to identify interventions that are most likely to succeed for a given individual. A series of co-creation workshops will translate these intervention models into tangible products, guided by older adults’ needs. Our risk profiling and intervention technologies will be implemented with community and industry partners to improve the efficacy of existing programs, exploit new pathways for intervention, and aid in the development of commercialization opportunities. Through the incorporation of older adults’ perspectives and the use of innovative analytical approaches, we will identify and implement innovative interventions for better mental health.

**Lay summary**

**Title: Personalized mental health care for seniors**

One in five seniors suffers from some form of mental illness. Lack of attention and support for mental illness not only harms the person, but also costs the healthcare system and economy billions.

Our challenge is to identify:

1. What seniors believe is good mental health
2. Factors that make and keep a person mentally healthy

Our objectives are to:

1. Understand seniors’ definitions of good mental health
2. Identify who is more likely to be at risk and/or showing early signs of poor mental health
3. Link people with help and support specific to their needs

Our project will use detailed information about the lives of thousands of people, collected in large study of aging, and advanced computer programming, such as machine learning. Rather than using traditional statistics that are only able to determine how a few variables are related to another, we will use machine learning to determine how many variables interact and relate to mental health. This will lead to the development of an “early-warning system” identifying seniors at risk and connecting them to personalized pathways for better mental health.

# References

1. Duke M. How to write a lay summary - digital curation centre “working level” guide. Edinburgh; 2012. Available from: http://www.dcc.ac.uk/resources/how-guides.pdf. Accessed 24 Nov 2019.

2. Kuehne LM, Olden JD. Opinion: Lay summaries needed to enhance science communication. Proc Natl Acad Sci. 2015;112:3585–6.

3. Smith M, Ashmore C. The lay summary in medical research proposals – is it becoming more important? 2010. Available from: https://www.researchgate.net/publication/262484948_The_Lay_Summary_in_medical_research_proposals_-_is_it_becoming_more_important. Accessed 25 Nov 2019.

4. Alzheimer’s Research UK. Writing a good lay summary of your research. Available from: https://www.alzheimersresearchuk.org/. Accessed 30 Sep 2019.

5. Sroka-Saidi K, Boggetti B, Schindler TM. Transferring regulation into practice: the challenges of the new layperson summary of clinical trial results. Med Writ. 2015;24:24–7.

6. Multiple Sclerosis Society of Canada. Guide to writing a lay summary. 2013. Available from: https://mssociety.ca/uploads/files/guide-to-writing-lay-summary-eng-final20130726.pdf. Accessed 24 Nov 2019.

7. Dubé CE, Lapane KL. Lay abstracts and summaries: writing advice for scientists. J Cancer Educ. 2014;29:577–9.

8. Haughton M, Machin D. The prevalence and characteristics of lay summaries of published journal articles. 2017. Available from: https://www.costellomedical.com/wp-content/uploads/2018/10/The-Prevalence-and-Characteristics-of-Lay-Summaries-of-Published-Journal-Articles.pdf. Accessed 25 Nov 2019.

9. Salita JT. Writing for lay audiences. Med Writ. 2015;24:183–9.

10. Cramm H, Breimer J, Lee L, Burch J, Ashford V, Schaub M. Best practices for writing effective lay summaries. J Mil Veteran Fam Heal. 2017;3:7–20.

11. Singh N, Vasudha. Writing lay summaries what medical writers need to know. Med Writ. 2018;27:49–54.

12. James LC, Bharadia T. Lay summaries and writing for patients: where are we now and where are we going? Med Writ. 2019;28:46–51.

13. Reeves A. Time to make it shorter: plain English in our context. Med Writ. 2015;24:4–8.

14. Brauburger BK, Sroka-saidi K, Schindler TM. New European clinical trial regulation: the requirement for lay summaries and its impact on medical communicators. AMWA J. 2015;30:60–3.
